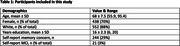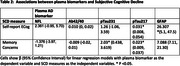# Plasma pTau and GFAP are associated with subjective cognitive decline and memory concerns in the remote, digital Brain Health Registry cohort

**DOI:** 10.1002/alz70857_101115

**Published:** 2025-12-24

**Authors:** Joseph Eichenbaum, Miriam T. Ashford, Chengshi Jin, Adam Diaz, Henrik Zetterberg, Kaj Blennow, Nicholas Ashton, Melanie J. Miller, Erika Cavallone, Juliet Fockler, Diana Truran‐Sacrey, Scott R. Mackin, Michael S. W. Weiner, Rachel L. Nosheny

**Affiliations:** ^1^ University of California, San Francisco, San Francisco, CA, USA; ^2^ San Francisco Veterans Affairs Medical Center, San Francisco, CA, USA; ^3^ Veterans Affairs Medical Center, San Francisco, CA, USA; ^4^ Northern California Institute for Research and Education (NCIRE), San Francisco, CA, USA; ^5^ University of Gothenburg, Mölndal, Sweden; ^6^ Department of Psychiatry and Neurochemistry, Institute of Neuroscience and Physiology, The Sahlgrenska Academy, University of Gothenburg, Mölndal, Sweden

## Abstract

**Background:**

Plasma‐based biomarkers have recently shown great potential for identifying AD in early symptomatic stages. Subjective Cognitive Decline (SCD), defined as self‐report of worsening cognition, may also be an early symptomatic stage on the AD clinical continuum. However, little is known about how plasma biomarkers identify AD pathology in the SCD population. Successful remote collection of plasma and self‐report data from a large, geographically distributed cohort demonstrates the potential for highly scalable approaches to screening and monitoring ADRD. We investigated the relationship between plasma AD biomarker levels and data collected in the Brain Health Registry (BHR), including both objective and subjective measures.

**Method:**

BHR participants were invited to join a remote plasma collection study. 629 participants (55+ and within 100 miles of a qualified Quest phlebotomy center, *Table 1*) completed a remote venipuncture blood draw. Plasma was analyzed by single molecular array for neurofilament light (NFL), amyloid β 42/40 (Aβ42/40), pTau231, pTau217 (ALZpath), and glial fibrillary acidic protein (GFAP). Linear regression was used to estimate associations between plasma biomarker levels and (1) Everyday Cognition Scale scores (ECog; a measure of subjective cognitive change); (2) self‐report subjective memory concerns; and (3) Performance on the Cogstate Brief Battery. All models covaried for age, gender, and education.

**Result:**

Higher pTau217 and pTau231 were associated with self‐report of a memory concern. Higher pTau217 and GFAP were associated with greater self‐reported decline on ECog (*Table 2*). There were no associations found with Aβ42/40, NFL, GFAP and self‐report of memory concern. Finally, there were no significant associations between any plasma biomarkers and Cogstate Brief Battery performance.

**Conclusion:**

The results suggest that remote, unsupervised, digital measures of SCD and subjective memory concern may be useful to help efficiently identify older adults with elevated plasma pTau and GFAP, who are at risk for cognitive decline and impairment due to AD. This approach can be scaled up to facilitate recruitment for clinical trials and AD observational studies, to identify suitable candidates for AD therapeutics, and for screening in clinical care settings.